# Why Do People Die of Anaphylaxis?—A Clinical Review

**DOI:** 10.1080/17402520500376277

**Published:** 2005-12

**Authors:** Arvind Kumar, Suzanne S. Teuber, M. Eric Gershwin

**Affiliations:** Division of Rheumatology, Allergy and Clinical Immunology Department of Internal Medicine University of California at Davis School of Medicine Davis CA USA

## Abstract

Anaphylaxis is a source of anxiety for patients and healthcare providers. It is a medical emergency that presents with a broad array of symptoms and signs, many of which can be deceptively similar to other diseases such as myocardial infarction, asthma, or panic attacks. In addition to these diagnostic challenges, anaphylaxis presents management difficulties due to rapid onset and progression, lack of appropriate self-treatment education and implementation by patients, severity of the allergic response, exacerbating medications or concurrent disease, and unpredictability. The most common causes of anaphylaxis are food allergies, stinging insects and immunotherapy (allergy shots) but idiopathic anaphylaxis, latex allergy and drug hypersensitive all contribute to the epidemiology. Reactions to IVP and other dyes are coined anaphylactoid reactions but have identical pathophysiology and treatment, once the mast cell has been degranulated. As many antigens can be the trigger for fatal anaphylaxis, it is useful to examine the features of each etiology individually, highlighting factors common to all fatal anaphylaxis and some specific to certain etiologies. Generally what distinguishes a fatal from non fatal reaction is often just the rapidity to apply correct therapy. Prevention is clearly the key and should identify high-risk patients in an attempt to minimize the likely of a severe reaction. Although fatal anaphylaxis is rare, it is likely underreported.


*Clinical & Developmental Immunology*, December 2005; 12(4): 281–287

Taylor & Francis

Taylor & Francis Group

### Why do people die of anaphylaxis?—A clinical review

ARVIND KUMAR, SUZANNE S. TEUBER & M. ERIC GERSHWIN

*Division of Rheumatology, Allergy and Clinical Immunology, Department of Internal Medicine, University of California at Davis School of Medicine, Davis, CA, USA*

**Abstract**
Anaphylaxis is a source of anxiety for patients and healthcare providers. It is a medical emergency that presents with a broad array of symptoms and signs, many of which can be deceptively similar to other diseases such as myocardial infarction, asthma, or panic attacks. In addition to these diagnostic challenges, anaphylaxis presents management difficulties due to rapid onset and progression, lack of appropriate self-treatment education and implementation by patients, severity of the allergic response, exacerbating medications or concurrent disease, and unpredictability. The most common causes of anaphylaxis are food allergies, stinging insects and immunotherapy (allergy shots) but idiopathic anaphylaxis, latex allergy and drug hypersensitive all contribute to the epidemiology. Reactions to IVP and other dyes are coined anaphylactoid reactions but have identical pathophysiology and treatment, once the mast cell has been degranulated. As many antigens can be the trigger for fatal anaphylaxis, it is useful to examine the features of each etiology individually, highlighting factors common to all fatal anaphylaxis and some specific to certain etiologies. Generally what distinguishes a fatal from non fatal reaction is often just the rapidity to apply correct therapy. Prevention is clearly the key and should identify high-risk patients in an attempt to minimize the likely of a severe reaction. Although fatal anaphylaxis is rare, it is likely underreported.

**Keywords:** *Allergy shots, food allergy, immunotherapy, latex, stinging insects*

### Introduction

The term "anaphylaxis" entered the scientific literature in 1902, when Richet and Potier described the hypotensive effects of sea anemone allergens in dogs (Cohen and Zelaya-Quesada 2002). Today, anaphylaxis generally refers to a potentially fatal group of symptoms and signs due to an immediate hypersensitivity reaction affecting one or multiple organ systems. Classically, anaphylaxis implies an immunoglobulin E (IgE)-mediated release of mediators from mast cells and basophils after antigen causes cross-linking of the IgE receptors on these cells. However, there is not a universally accepted definition of anaphylaxis, and it is often used to describe non-IgE-mediated or other non-immunologic events (sometimes called "anaphylactoid") as well (Anthony 2005). Symptoms and signs that may be present singly, in combination, and in varying degrees of severity involve the skin (urticaria, angioedema, flushing, pruritis), the cardiopulmonary system (hypotension, syncope/presyncope, tachycardia, arrhythmia, wheezing, stridor, hypoxia, sudden death), the gastrointestinal system (nausea, vomiting, diarrhea, abdominal pain) and other systems (uterine cramps, rhinoconjunctivitis, headache, sense of impending doom) (Table I). The lessons gleaned from reviews of cases of fatal anaphylaxis may help physicians understand why people die and may help initiate management approaches that prevent anaphylaxis. With this in mind, the goal of this discussion is to highlight some of the significant findings and lessons learned from studies on fatal anaphylaxis.

### Epidemiology

Exact incidence measures for anaphylaxis and fatal anaphylaxis are unknown, and many studies document under-reporting of events (Lieberman 2003). This would suggest that the data we have, underestimates the true incidence of this problem. Numerous authors have

Correspondence: M. E. Gershwin, Division of Rheumatology, Allergy and Clinical Immunology, University of California at Davis School of Medicine, 451 E. Health Sciences Drive, Suite 6510, Davis, CA 95616, USA. Tel: 1 530 752 2884. Fax: 1 916 734 3402. E-mail: megershwin@ucdavis.edu

ISSN 1740-2522 print/ISSN 1740-2530 online © 2005 Taylor & Francis
DOI: 10.1080/17402520500376277

Table I. Signs and symptoms of anaphylaxis in the various target organs and tissues.

| Affected organ or tissue | Specific signs and symptoms | Percentage of patients affected: Yocum et al. adults ($n = 133$) | Percentage of patients affected: Dibs et al. children 1–19 years of age ($n = 55$) |
|---|---|---|---|
| Cutaneous/dermatologic | Urticaria | 100 | 93 |
| | Angioedema | | |
| | Pruritus | | |
| | Flushing | | |
| | Conjunctivitis or chemosis | | |
| Respiratory | *Upper airway:* | 69 | 93 |
| | Nasal congestion | | |
| | Sneezing | | |
| | Hoarseness | | |
| | Stridor | | |
| | Cough | | |
| | Obstruction | | |
| | Throat tightness | | |
| | Laryngeal edema | | |
| | *Lower airway:* | | |
| | Dyspnea | | |
| | Bronchospasm | | |
| | Tachypnea | | |
| | Wheezing | | |
| | Cyanosis | | |
| | Respiratory arrest | | |
| Oral/gastrointestinal | Intraoral | 24 | 13 |
| | Angioedema | | |
| | Emesis | | |
| | Nausea | | |
| | Abdominal cramps | | |
| | Dysphagia | | |
| | Diarrhea | | |
| Cardiovascular | Tachycardia | 41 | 26 |
| | Presyncope | | |
| | Hypotension | | |
| | Shock | | |
| | Chest pain | | |
| | Bradycardia | | |
| | Orthostasis | | |
| | Cardiac arrest | | |
| Neurological | Feeling of impending doom | Not determined | 26 |
| | Dizziness | | |
| | Weakness | | |
| | Syncope | | |
| | Seizures | | |

collected data in reviews of anaphylaxis and its epidemiology (Kemp and Lockey 2002, Lieberman 2003, Moneret-Vautrin et al. 2005). Despite the limitations and variability of the data available, some sense of the scale of the problem is obtained by looking at estimates of incidence of fatal anaphylaxis. An international epidemiological study estimates that 154 fatal episodes of anaphylaxis occur per 1,00,000 hospitalized patients per year (International collaborative study of severe anaphylaxis 1998). In the United States, the risk of anaphylaxis per individual is suggested to be from less than one, up to 3% (Yocum et al. 1999, Kemp and Lockey 2002), while 1500 deaths are thought to occur annually due to anaphylaxis (Neugut et al. 2001). Pumphrey has collected detailed data on anaphylactic deaths in the United Kingdom (UK) from 1992–2001. His data suggests that in the UK, there are about 20 fatalities per year, which corresponds to one per year for each 3 million people (Pumphrey 2004). Unfortunately this is likely an underestimate as many fatalities go unreported in the medical literature.

### Pathology and modes of death

Autopsy data from patients who died from anaphylaxis reveal that, in many cases, there are no specific findings by macroscopic exam (Pumphrey and Roberts 2000). Most fatal reactions are limited to a single system or area (such as cardiovascular or respiratory or upper airway), not usually the case with less severe anaphylaxis (Pumphrey 2004). Pumphrey found that shock occurred from different causes in older versus younger patients. Younger patients who died of anaphylactic shock tended to experience vasodilation with hypotension, leading to pulseless electrical activity and death (Pumphrey 2004). In the older patients in his UK register, shock was found to be from arrhythmia, which is more commonly found in older hearts with pre-existing disease (Pumphrey 2004). In this series, respiratory arrest was due to bronchospasm, upper airway edema, or a combination of the two. Bronchospastic respiratory arrest was more common in food-induced anaphylaxis, while shock was more likely with drugs and venom reactions. Respiratory arrest due to upper airway edema was more common in food and venom reactions than in drug reactions (Pumphrey 2004). Less common modes of death include disseminated intravascular coagulation and complications from epinephrine overdose. One practical point related to modes of death in fatal anaphylaxis relates to posture. Pumphrey found that four of the patients in his register that died of shock died within seconds of a "change to a more upright posture" (Pumphrey 2003). It is felt that patients suffering from anaphylactic shock cannot tolerate the emptying of the vena cava that results when they are made to sit up or stand since it will cause loss of left ventricular filling pressure, myocardial ischemia, pulseless electrical activity, and quickly, death (Pumphrey 2003). The lesson from these facts is that patients with anaphylaxis should be kept supine if their respiratory status will tolerate this. They should also have their legs elevated to increase filling of the vena cava and heart. These recommendations should be incorporated into education for patients and medical staff (Pumphrey 2003).

### Etiology

The most commonly identified causes of anaphylaxis are immunotherapy, foods, medications, diagnostic agents, stinging insect venom and latex (Lieberman 2003). Idiopathic anaphylaxis is also significant, since studies have shown that the etiology of anaphylaxis is not found in 47% or more of cases presenting to allergist-immunologists (Kemp et al. 1995, Lieberman 2003). As any of the above can be the trigger for fatal anaphylaxis, it is useful to examine the features of each etiology individually, highlighting factors common to all fatal anaphylaxis and some specific to certain etiologies. Reactions to IVP and other contrast media are identical to anaphylaxis, once the mast cell has degranulated and will not be discussed in further detail except to emphasize that what distinguished a fatal from not fatal reaction is often just the rapidity to apply correct therapy.

### Fatal anaphylaxis by etiology

### *Food anaphylaxis*

Several series, epidemiological studies, and literature reviews report food as the most common cause of anaphylaxis (Yocum and Khan 1994, Kemp et al. 1995, Yocum et al. 1999, Lieberman 2003). It is estimated that foods cause fatal anaphylaxis in 125–150 Americans annually (Burks et al. 1999, Bock et al. 2001). Pumphrey found that about 6 of 19 anaphylactic fatalities per year that occur in the UK are due to foods (Pumphrey). Peanuts and tree nuts are the predominant offenders, accounting for 94% of 32 fatal cases reported to a national registry and making up the majority of food anaphylaxis deaths in Pumphrey's UK register of fatal anaphylaxis (Bock et al. 2001). However, all foods (Moneret-Vautrin et al. 2005) and allergens have the potential to cause fatal anaphylaxis. Fatalities found in the UK register also include 14% due to cow's milk, and smaller numbers from fish, shellfish, chickpea, hen's egg, and banana (Pumphrey 2004). Bock et al. (2001) found that food-related fatal anaphylaxis affects both sexes equally, while the UK register described a female predominance (Pumphrey 2004). The majority of victims are young (adolescents to young adults) (Sampson et al. 1992, Wuthrich 2000, Bock et al. 2001, Pumphrey 2004). All but one of the 32 patients in Bock's study and all of the patients with food as the etiology in the UK register had a history of a prior reaction to foods before the fatal event (Pumphrey and Nicholls 2000, Bock et al. 2001). Of even more significance is the fact that 80% of the UK registry patients who died of food-related anaphylaxis had only minor previous reactions, suggesting that severity of prior reaction cannot be used to identify who is at risk of fatal reactions, or even to guide prescription of epinephrine self-treatment (Pumphrey 2004). Asthma seems to play an important role in fatal food anaphylaxis and may make reversing bronchospasm from anaphylaxis more difficult (Sampson et al. 1992). All but one in Bock's study had asthma (Bock et al. 2001), and all of the those with food as a cause in the UK register had difficulty breathing, with 86% of these proceeding to respiratory arrest (Pumphrey 2003). Most of the UK register patients who died due to foods also had suboptimally controlled asthma, many using daily beta-agonist therapy and not regularly using inhaled corticosteroids (Pumphrey 2004). Frequent beta-agonist use may result in resistance to epinephrine, making treatment of anaphylaxis more difficult (Pumphrey and Nicholls 2000). The predominance of pulmonary symptoms in these cases caused some paramedics to be uncertain about initiating an anaphylaxis treatment protocol or one for asthma, which led to treatment delays or inappropriate treatments, both of which may have contributed to the fatal outcomes (Pumphrey and

Nicholls 2000). Based on these data, Pumphrey and Nicholls (2000) argues that we may decrease food anaphylaxis fatalities by emphasizing use of inhaled beta-agonists (as a supplement to epinephrine) as part of the acute anaphylaxis self-treatment plan. In fatalities due to foods, although the median time to respiratory or cardiac arrest was 30 min in the UK register, some food reactions started with very mild symptoms and progressed over several hours to a final rapid arrest (Pumphrey and Nicholls 2000). Such initially mild symptoms may lead to less aggressive management that allows progression of ultimately fatal reactions. Another problem that contributes to fatal results in food-related anaphylaxis is delayed or absent treatment with epinephrine. Ninety percent of those in Bock's study did not have epinephrine auto-injectors (EAIs) (Bock et al. 2001). Other studies have also found that many patients who have a prescribed EAI do not carry or do not use the device in situations where EAI use was thought to be appropriate by the physician (Pumphrey and Nicholls 2000). In some instances, EAIs are not used due to lack of appropriate training (Huang 1998). In others, the device is expired (Pumphrey and Nicholls 2000). In many cases of food-anaphylaxis fatalities in the UK registry, there was a delay in epinephrine treatment, with many not receiving this treatment until after cardiopulmonary arrest (Pumphrey and Nicholls 2000). This was due to the rapid evolution of the reaction in some and the availability of treatment in others. Although, early use of epinephrine in anaphylaxis is supported by national management guidelines (Anthony 2005) and experts in the field (Bock et al. 2001), sometimes fatalities occur despite appropriately timed and dosed use of epinephrine. This was seen in both the UK registry and the group studied by Bock et al. (2001) (Pumphrey and Nicholls 2000). This data makes education of patients regarding allergen avoidance, food labels, cross-contamination, and early recognition of the symptoms and signs of anaphylaxis all the more important.

Given the high incidence of cross-contamination with nuts, particularly when eating in restaurants, some suggest that any patient allergic to one nut should be evaluated for allergy to other nuts and be advised to avoid all nuts (Pumphrey and Nicholls 2000). Other issues that make fatal reactions to food more likely include the minute quantities (Hourihane et al. 1997) of allergen sometimes required to cause anaphylaxis and the resultant difficulties in completely avoiding these allergens. Despite education of parents and patients, unintentional exposures to food allergens, particularly nuts, occur and may cause recurrent reactions (Sicherer et al. 1998). This fact again emphasizes the need for education of patients and physicians regarding action plans for food anaphylaxis and early recognition of symptoms.

*Drug/diagnostic agent/iatrogenic anaphylaxis*

Iatrogenic causes of anaphylaxis are common. Drugs are felt to be second only to foods as a cause for anaphylaxis (Lieberman 2003), and hundreds of these agents have been known to cause anaphylaxis (Neugut et al. 2001). These reactions affect older patients than due foods (Pumphrey). Again, incidence data is variable by study, 50 deaths annually in the Netherlands (van der Klauw et al. 1996) per year, 30 cases over a 22-year period in Denmark (Lenler-Petersen et al. 1995), and 9 per year (50% of total deaths) in the UK registry (Pumphrey). Antibiotics, particularly beta-lactams, are felt to be the most frequent offenders with NSAIDS also causing significant numbers of anaphylaxis episodes (Lieberman 2003). Radiocontrast media is a common fatal trigger, causing an estimated 900 deaths annually in the United States (Neugut et al. 2001). Lower-osmolarity, non-ionic agents appear to be safer (Katayama et al. 1990). Risk of reaction in patients on beta-blockers is higher (Toogood 1988). During anesthesia, common causes of fatal anaphylaxis include muscle relaxants, which may have a synergistic response with opioids to make reactions more severe (Pumphrey 2004). Allergen immunotherapy has been found to cause fatal reactions in 1 per every 2.5 million shots given, and causes 3.4 deaths annually. The deaths are primarily in asthmatics with sub-optimal control of asthma, during maintenance dosing of immunotherapy, in settings without adequate mechanisms of anaphylaxis treatment, and associated with delayed administration of epinephrine, but not typically due to dosing errors (Bernstein et al. 2004). The same study reported one death from allergen skin testing to 90 different food allergens performed on a patient with poorly controlled asthma. Although, latex allergy has increased over the last decade, latex-induced fatal anaphylaxis seems to be rare (Pumphrey 2004, Bernstein et al. 2004, Moneret-Vautrin et al. 2005).

Drugs usually cause cardiac arrest with shock rather than asphyxia from bronchospasm or laryngeal/pharyngeal edema, which is seen more commonly with food cases (Pumphrey and Nicholls 2000). Also, deaths from drug-induced anaphylaxis tend to be characterized by a much more rapid (within minutes) onset and progression to arrest (Pumphrey and Nicholls 2000). Such rapid progression of symptoms can make treatment more difficult and error-prone. Pumphrey and others have documented that epinephrine, arguably the most important drug in anaphylaxis management, is sometimes given at inappropriately high doses intravenously during anaphylaxis (Pumphrey and Nicholls 2000). This has caused fatal pulmonary edema and myocardial infarction. Clearly, further education of physicians and nurses regarding giving only dilute epinephrine doses intravenously is warranted. Other efforts that may prevent these errors include regular anaphylaxis drills in clinic and hospital settings. The fact that these deaths often occur despite aggressive and rapid treatment for hospitalized patients makes it clear that some reactions are so severe that death is not preventable. In such cases, measures to avoid agents that may have cross-reactive potential may be at least as important as rescue efforts (Pumphrey and Nicholls 2000).

*Stinging insect venom anaphylaxis*

Venom reactions comprise 11–58% of anaphylaxis triggers, dependent on which study is cited (Moneret-Vautrin et al. 2005). An estimated 40–100 Americans die annually due to envenomation and subsequent anaphylaxis from stinging insects of the order hymenoptera (Neugut et al. 2001). One quarter of the 20 patients that die from anaphylaxis in the UK annually die from insect venom reactions (Toogood 1988). The patients in the UK registry who died from venom reactions had a peak age of 45–70, tended to be male, and were often atopic (Pumphrey 2004). Time to arrest in this group was 10–15 min, intermediate relative to food and drug cases. These patients also tended to die due to shock, similar to drug cases and unlike the deaths related to foods. Another disturbing feature of the group is that more than two-thirds of these venom deaths occurred in patients who had never had a generalized reaction to venom in the past, and thus were not even candidates for carrying EAI or for consideration of venom immunotherapy. Additionally, some venom deaths occur in patients who have not received immunotherapy, but who should have received it according to current guidelines (Pumphrey 2004). This makes it clear that at minimum, we should be offering this generally very effective form of therapy to patients who meet desensitization criteria and lack definite contraindications to treatment. Some (Sasvary and Muller 1994) have found a higher incidence of coronary artery disease in those that die from venom reactions, but others (Pumphrey 2004) have not. The fact that some deaths (Light 2001) have occurred in patients despite venom immunotherapy, emphasizes the need for effective education of patients regarding avoidance, regular carrying of EAIs, and proper EAI use. Another feature of venom deaths is its relationship to mastocytosis. The UK register documented one case (Pumphrey 2004), and it is known that patients with mastocytosis have an elevation of tryptase at baseline that may lead to more severe reactions to venom (Ludolph-Hauser et al. 2001, Haeberli et al. 2003).

*Immunotherapy*

Even though the risk of fatal reactions to immunotherapy is low, any death is unacceptable. Further deaths from immunotherapy are nearly always the result of error. Therefore, many investigators have looked for commonalities between fatal cases in an attempt to identify specific risk factors. Since most deaths after immunotherapy are attributable to fatal anaphylaxis, the most severe form of a systemic allergic reaction (SR), great efforts have also been made to identify risk factors for SRs in general and for severe, but non-fatal, SRs in particular. Unfortunately, widely differing definitions of SR have been used and this is reflected in the great variation in the reported rates of SRs, which range from $<1$ to 100% of patients. Factors like differences in patient characteristics, types of allergens, and administration protocols are also likely to contribute to the variability of the results. Some of these risk factors include the presence of asthma; multiple studies have reported a higher proportion of asthma patients among the deaths associated with immunotherapy (Medicines CoS 1986, Lockey et al. 1987, Reid et al. 1993, Kordash and Miller 1997). In addition, many fatal reactions to immunotherapy occur when the doses are increased, as opposed to maintenance. However, it should be emphasized that maintenance dose should not contain an excessive dose of allergen (Medicines CoS 1986, Tabar et al. 1993, Nettis et al. 2002). It has also been noted that anaphylaxis to pollen allergens is likely to occur during allergy season when patients are also exposed to aeroallergen. It is also noted that patients with high levels of IgE, as noted by the skin test or RAST, are also more commonly affected by fatal reactions (Lin et al. 1993, Bousquet et al. 1998). Although at least one paper has suggested that local reactions may not be predictive of fatal reactions, it should be emphasized that multiple other observations have suggested that local reactions are a major risk factor and while not 100% predictive, are a serious warning that allergen immunotherapy dosage should be reduced. Fatal reactions have also been reported in patients who had previously undergone serious

Table II. Sources of potential treatment errors in immunotherapy (IT).

1. Errors in dosage, including failure to reduce the dosage after a longer than scheduled interval between injections or after an adverse reaction to a previous injection or when injecting extract from a new vial, particularly one from a different manufacturer.
2. Failure to provide the patients with all the appropriate information.
3. Failure to observe the patient for the appropriate length of time after the injection.
4. Mixture of seasonal and perennial allergens.
5. Failure to adhere to established guidelines for contraindications to IT.
6. Failure to postpone the injection because of an existing infection or asthma exacerbation.
7. Inadvertent intravenous administration.
8. Administration of the wrong extract.

Table III. Treatment of anaphylaxis.

1. Symptoms not immediately life-threatening may rapidly progress unless treated promptly
   (a) Intramuscular or subcutaneous epinephrine (intramuscular preferred)
      (i) Adults-0.2 ml (0.2 mg)–0.5 ml (0.5 mg) of a 1:1000 w/v (1 mg/ml) dilution every 10–15 min, up to a maximum of 1 ml (1 mg) per dose
      (ii) Children-0.01 ml (0.01 mg)/kg body weight up to a maximum of 0.5 ml (0.5 mg) per dose of 1:1000 (1 mg/ml) w/v dilution, repeated every 15 min for 2 doses, then every 4 h as needed
   (b) Diphenhydramine: 1–2 mg/kg or 25–50 mg per dose may be given parenterally.
   (c) Intravenous Corticosteroids (hydrocortisone (e.g. Solucortef)): Recommended, but efficacy not clearly delineated.
2. Immediate treatment of life-threatening anaphylaxis includes:
   (a) Intramuscular or subcutaneous epinephrine at dosage described above
   (b) Intravenous epinephrine can be considered if the patient significantly worsens despite repeated doses of subcutaneous or intramuscular epinephrine; administered either by using a formulation of 1:10,000 (0.1 mg/ml) or 1:1,00,000 (0.01 mg/ml) w/v dilution initially titrated at 1 μg/min which can be increased to 2–10 μg/min as needed
   (c) CPR as needed to restore circulation and respiration
   (d) Vasopressors and intravenous fluids to treat hypotension
   (e) Oxygen
   (f) Inhaled β-adrenergic bronchodilator for bronchospasm
   (g) Constant monitoring of cardiac, respiratory and circulatory systems.
3. A good clinical response indicates resolution of the anaphylactic reaction. Continued monitoring is indicated if (1) the response appears incomplete, or (2) biphasic anaphylaxis appears likely. Additional history and laboratory tests will add to the diagnostic base. Consider antihistamines for urticaria/angioedema, and $H_2$-receptor blocking drugs for epinephrine-resistant hypotension.
4. Additional treatment may be required for patients who:
   (1) are unresponsive to epinephrine due to β-adrenergic blocking therapy (consider fluid replacement, epinephrine, and if still unresponsive consider glucagon 1 mg intravenously as a bolus with continuous infusion of 1–5 mg/h)
   (2) for prominent respiratory symptoms (consider inhaled β-adrenergic agonist)
   (3) need hospital admission for extended management or management of comorbid conditions.
5. Monitor for late-phase reactions
   (1) in a medical setting for an extended period, for life-threatening episodes, or
   (2) at home if the reaction was mild.
6. Follow-up includes complete evaluation and a long-term treatment plan in consultation with an allergist/immunologist.

The diagnosis and management of anaphylaxis. J Allergy Clin Immunol 1998;101:S465–S528.

systemic reactions to immunotherapy, in patients in whom a new vial of allergen has been opened, and in patients receiving beta-blocking agents (Lockey et al. 1987, Malling and Weeke 1993, Reid et al. 1993, Bousquet et al. 1998). We should note that recent studies suggest that beta-blockers, particularly the more selective agents, may not pose a significant risk (Hepner et al. 1990). Fatal reactions also occur from treatment errors (Table II). Immunotherapy should never be undertaken unless physicians are aware of and aggressive in the correct treatment of anaphylaxis.

### *Idiopathic anaphylaxis*

As discussed previously, no cause is found in many cases of anaphylaxis. Idiopathic anaphylaxis is defined as anaphylaxis for which no identifiable cause is found. Unidentified food allergens are thought to be the trigger in some of these cases (Pumphrey 2004). Despite the frequency of this diagnosis, fatalities due to the syndrome of idiopathic anaphylaxis are uncommon. One series of 350 patients had one fatality (Krasnick et al. 1996), though others also have reported some cases (Patterson et al. 1995).

### Final comments

Anaphylaxis is a source of anxiety for patients and healthcare providers. It is a medical emergency that presents with a broad array of symptoms and signs, many of which can be deceptively similar to other diseases such as myocardial infarction, asthma, or panic attacks. In addition to these diagnostic challenges, anaphylaxis presents management difficulties due to rapid onset and progression, lack of appropriate self-treatment education and implementation by patients, severity of the allergic response, exacerbating medications or concurrent disease, and unpredictability. Large randomized clinical trials regarding the best management are unlikely to be performed given the rarity of anaphylaxis and ethical issues regarding withholding specific treatments in these potentially fatal situations. It is likely that many of us as physicians will face severe or even fatal anaphylaxis in patients due to foods, drugs, or venom. Clearly there is no way to eliminate fatal reactions in all cases, but attention to some of the lessons learned from prior deaths may decrease the frequency of these tragic episodes (Table III).

### References

Lieberman P, Kemp S, et al. 2005. The diagnosis and management of anaphylaxis: An updated practice parameter. J Allergy Clin Immunol 115:S483–S523.

Bernstein DI, Wanner M, Borish L, Liss GM. 2004. Twelve-year survey of fatal reactions to allergen injections and skin testing: 1990–2001. J Allergy Clin Immunol 113:1129–1136.

Bock SA, Munoz-Furlong A, Sampson HA. 2001. Fatalities due to anaphylactic reactions to foods. J Allergy Clin Immunol 107:191–193.

Bousquet J, Czarlewski W, Cougnard J, Danzig M, Michel FB. 1998. Changes in skin-test reactivity do not correlate with clinical efficacy of H1-blockers in seasonal allergic rhinitis. Allergy 53:579–585.

Burks W, Bannon GA, Sicherer S, Sampson HA. 1999. Peanut-induced anaphylactic reactions. Int Arch Allergy Immunol 119:165–172.

Cohen SG, Zelaya-Quesada M. 2002. Portier, Richet, and the discovery of anaphylaxis: A centennial. J Allergy Clin Immunol 110:331–336.

Haeberli G, Bronnimann M, Hunziker T, Muller U. 2003. Elevated basal serum tryptase and hymenoptera venom allergy: Relation to severity of sting reactions and to safety and efficacy of venom immunotherapy. Clin Exp Allergy 33:1216–1220.

Hepner MJ, Ownby DR, Anderson JA, Rowe MS, Sears-Ewald D, Brown EB. 1990. Risk of systemic reactions in patients taking beta-blocker drugs receiving allergen immunotherapy injections. J Allergy Clin Immunol 86:407–411.

Hourihane JB, Kilburn SA, Nordlee JA, Hefle SL, Taylor SL, Warner JO. 1997. An evaluation of the sensitivity of subjects with peanut allergy to very low doses of peanut protein: A randomized, double-blind, placebo-controlled food challenge study. J Allergy Clin Immunol 100:596–600.

Huang SW. 1998. A survey of epi-PEN use in patients with a history of anaphylaxis. J Allergy Clin Immunol 102:525–526.

International collaborative study of severe anaphylaxis. 1998. An epidemiologic study of severe anaphylactic and anaphylactoid reactions among hospital patients: Methods and overall risks. Epidemiology 9:141–146.

Katayama H, Yamaguchi K, Kozuka T, Takashima T, Seez P, Matsuura K. 1990. Adverse reactions to ionic and nonionic contrast media. A report from the Japanese committee on the safety of contrast media. Radiology 175:621–628.

Kemp SF, Lockey RF. 2002. Anaphylaxis: A review of causes and mechanisms. J Allergy Clin Immunol 110:341–348.

Kemp SF, Lockey RF, Wolf BL, Lieberman P. 1995. Anaphylaxis. A review of 266 cases. Arch Intern Med 155:1749–1754.

van der Klauw MM, Wilson JH, Stricker BH. 1996. Drug-associated anaphylaxis: 20 years of reporting in the Netherlands (1974–1994) and review of the literature. Clin Exp Allergy 26:1355–1363.

Kordash T, Miller J. 1997. J Allergy Clin Immunol 99:S67.

Krasnick J, Patterson R, Meyers GL. 1996. A fatality from idiopathic anaphylaxis. Ann Allergy Asthma Immunol 76:376–378.

Lenler-Petersen P, Hansen D, Andersen M, Sorensen HT, Bille H. 1995. Drug-related fatal anaphylactic shock in Denmark 1968–1990. A study based on notifications to the committee on adverse drug reactions. J Clin Epidemiol 48:1185–1188.

Lieberman P. 2003. Anaphylaxis and anaphylactoid reactions. In: Adkinson Jr., N, editor. Middleton's allergy: Principles and practice. MO: Mosby. p 1497–1592.

Light WC. 2001. Insect sting fatality 9 years after venom treatment (venom allergy, fatality). J Allergy Clin Immunol 107:925.

Lin MS, Tanner E, Lynn J, Friday Jr, GA. 1993. Nonfatal systemic allergic reactions induced by skin testing and immunotherapy. Ann Allergy 71:557–562.

Lockey RF, Benedict LM, Turkeltaub PC, Bukantz SC. 1987. Fatalities from immunotherapy (IT) and skin testing (ST). J Allergy Clin Immunol 79:660–677.

Ludolph-Hauser D, Rueff F, Fries C, Schopf P, Przybilla B. 2001. Constitutively raised serum concentrations of mast-cell tryptase and severe anaphylactic reactions to hymenoptera stings. Lancet 357:361–362.

Malling H-J, Weeke B. 1993. Position paper: Immunotherapy. The European Academy of Allergology and Clinical Immunology. Allergy 48:7–35.

Medicines CoS 1986. Br Med J 293:948.

Moneret-Vautrin DA, Morisset M, Flabbee J, Beaudouin E, Kanny G. 2005. Epidemiology of life-threatening and lethal anaphylaxis: A review. Allergy 60:443–451.

Nettis E, Giordano D, Pannofino A, Ferrannini A, Tursi A. 2002. Safety of inhalant allergen immunotherapy with mass units-standardized extracts. Clin Exp Allergy 32:1745–1749.

Neugut AI, Ghatak AT, Miller RL. 2001. Anaphylaxis in the United States: An investigation into its epidemiology. Arch Intern Med 161:15–21.

Patterson R, Clayton DE, Booth BH, Greenberger PA, Grammer LC, Harris KE. 1995. Fatal and near fatal idiopathic anaphylaxis. Allergy Proc 16:103–108.

Pumphrey RS. 2003. Fatal posture in anaphylactic shock. J Allergy Clin Immunol 112:451–452.

Pumphrey RS. 2004. Fatal anaphylaxis in the UK, 1992–2001. Novartis Found Symp 257:116–128; discussion 28–32, 57–60, 276–285.

Pumphrey R. 2004. Anaphylaxis: Can we tell who is at risk of a fatal reaction? Curr Opin Allergy Clin Immunol 4:285–290.

Pumphrey RS, Nicholls JM. 2000. Epinephrine-resistant food anaphylaxis. Lancet 355:1099.

Pumphrey RS, Roberts IS. 2000. Postmortem findings after fatal anaphylactic reactions. J Clin Pathol 53:273–276.

Reid MJ, Lockey RF, Turkeltaub PC, Platts-Mills TA. 1993. Survey of fatalities from skin testing and immunotherapy 1985–1989. J Allergy Clin Immunol 92:6–15.

Sampson HA, Mendelson L, Rosen JP. 1992. Fatal and near-fatal anaphylactic reactions to food in children and adolescents. N Engl J Med 327:380–384.

Sasvary T, Muller U. 1994. Fatalities from insect stings in Switzerland 1978–1987. Schweiz Med Wochenschr 124:1887–1894.

Sicherer SH, Burks AW, Sampson HA. 1998. Clinical features of acute allergic reactions to peanut and tree nuts in children. Pediatrics 102:e6.

Tabar AI, Garcia BE, Rodriguez A, Olaguibel JM, Muro MD, Quirce S. 1993. A prospective safety-monitoring study of immunotherapy with biologically standardized extracts. Allergy 48:450–453.

Toogood JH. 1988. Risk of anaphylaxis in patients receiving beta-blocker drugs. J Allergy Clin Immunol 81:1–5.

Wuthrich B. 2000. Lethal or life-threatening allergic reactions to food. J Investig Allergol Clin Immunol 10:59–65.

Yocum MW, Khan DA. 1994. Assessment of patients who have experienced anaphylaxis: A 3-year survey. Mayo Clin Proc 69:16–23.

Yocum MW, Butterfield JH, Klein JS, Volcheck GW, Schroeder DR, Silverstein MD. 1999. Epidemiology of anaphylaxis in Olmsted County: A population-based study. J Allergy Clin Immunol 104:452–456.

